# Plasma ctDNA increases tissue NGS-based detection of therapeutically targetable mutations in lung cancers

**DOI:** 10.1186/s12885-023-10674-z

**Published:** 2023-03-31

**Authors:** Jianjiang Xie, Weishen Yao, Lingxiu Chen, Wenjun Zhu, Qiang Liu, Geng Geng, Jing Fang, Yang Zhao, Li Xiao, Zhenhua Huang, Jing Zhao

**Affiliations:** 1 Department of Thoracic surgery, School of Medicine, Guangzhou First People’s Hospital, South China University of Technology, Guangzhou, 510180 China; 2 Department of Cardiothoracic Surgery, Nanhai District People’s Hospital of Foshan, Foshan, 528200 China; 3 Department of Pulmonary and Critical Care Medicine, Three Gorges Hospital of Chongqing University, Chongqing, 404000 China; 4 Department of Oncology, Tongji Hospital, Tongji Medical College, Huazhong University of Science and Technology, Wuhan, 430030 P.R. China; 5 Shenyang Chest Hospital & Tenth People’s Hospital, Shenyang, Liaoning 110044 China; 6 Department of Thoracic and Cardiac Surgery, WuHu Hospital, East China Normal University, Wuhu, Anhui 241000 China; 7 Department of Oncology, School of Medicine, Zhongshan Hospital of Xiamen University, Xiamen, 361004 China; 8 Department of Thoracic Surgery, Shanghai Chest Hospital, Shanghai Jiao Tong University, Shanghai, 200030 China; 9 Department of Oncology, Nanfang Hospital of Southern Medical University, Guangzhou, 510515 China

**Keywords:** Lung cancer, Next generation sequencing, Plasma, Circulating tumor DNA

## Abstract

**Background:**

Circulating tumor DNA (ctDNA) has been becoming a novel convenient and noninvasive method for dynamically monitoring landscape of genomic information to guild personalized cancer treatment. In this study we comprehensively evaluated the additional value of plasma ctDNA to routine tissue next generation sequencing (NGS) of therapeutically targetable mutations in lung cancers.

**Methods:**

The tumor tissues and peripheral blood samples from 423 cases of patients with lung cancer were subjected to NGS of mutations in oncodrivers (*EGFR*, *ERBB2*, *ALK*, *ROS1*, *C-MET*, *KRAS*, *BRAF*, *RET*, *BRCA1* and *BRCA2*).

**Results:**

One hundred and ninety-seven cases showed both plasma and tissue positive and 96 showed both negative. The concordance for tissue and blood detection was 69.27% (293/423). 83 (19.62%) cases showed positive by tissue NGS alone and 47 (11.11%) positive by plasma ctDNA alone. The sensitivity of tissue and plasma detection was 85.63%, and 74.62%, respectively. Plasma had lower detection and sensitivity than tissue, but plasma additionally detected some important mutations which were omitted by tissue NGS. Plasma plus tissue increased the detection rate of 66.19% by tissue alone to 77.30% as well as the sensitivity of 85.63–100%. Similar results were also observed when the cases were classified into subpopulations according to different stages (IV vs. III vs. I-II), grades (low vs. middle grade) and metastatic status (metastasis vs. no metastasis).

**Conclusion:**

Plasma ctDNA shares a high concordance with tissue NGS, and plasma plus tissue enhances the detection rate and sensitivity by tissue alone, implying that the tissue and plasma detection should be mutually complementary in the clinical application.

**Supplementary Information:**

The online version contains supplementary material available at 10.1186/s12885-023-10674-z.

## Introduction

A recent global cancer investigation by the International Agency for Research on Cancer revealed that lung cancer ranks the second most diagnosed cancer and the main cause of cancer death in 2020, with an estimation of 2,200,000 new cancer cases (11.4%) and 1,800,000 cancer death in 2020 (18.0%) [1]. Lung cancer has a worse prognosis, with a 5-year survival of less than 18% [2]. Despite the advancement of various treatments, such as surgery, chemotherapy, radiotherapy, targeted therapy and immunotherapy, the outcomes and prognosis of lung cancer are still unsatisfactory, which may be due to high intra- and inter- tumoral genomic heterogeneity.

Tumor tissue-based next generation sequencing (NGS) of therapeutic targets is important for personalized precision treatment for lung cancer, while tumor tissues may not always be available (especially for advanced cancers with a poor clinical condition) or may be ineligible for NGS. Circulating tumor DNA (ctDNA) is a novel noninvasive method for obtaining landscape of genomic information to guide personalized cancer treatment that overcomes the effect of intratumor heterogeneity [3]. ctDNA has been rapidly using for molecular profiling, monitoring and prognostication of cancers [4, 5], with a good concordance with the matched tumor tissue NGS [6]. In lung cancer, plasma ctDNA has a satisfactory sensitivity in the identification of oncogenic mutations, with a high tissue concordance [7–10]. For example, Lin et al. showed that tissue NGS had a significantly higher sensitivity (identification of 74 mutations, 94.8%) of identifying the clinically relevant mutations in lung adenocarcinoma than plasma (41 mutations, 52.6%), with an overall concordance of 59%; tissue NGS exhibited significantly higher sensitivity and accuracy in comparison with plasma-NGS, regardless of assay in newly diagnosed vs. treated patients, and metastatic vs. nonmetastatic disease; plasma-NGS also plays an important role in detection of clinically relevant alterations, especially when tissue testing is not available, while with a low sensitivity, which means that a negative plasma-NGS result should be validated through a tissue-based assay [7]. Plasma comprehensive molecular profiling is reliable to detect oncogenic drivers in advanced non-small-cell lung cancers (NSCLCs), although it cannot replace tissue NGS [8]. However, these studies just involved inadequate cases or limited analysis.

Until now there have very few reports observing the additional value of plasma ctDNA to routine tissue NGS in the detection of clinically relevant mutations in large number of lung cancers, not only in whole population but also in subpopulations classified according to different stage, grades and metastatic status, yet. In this study we comprehensively observed the detective efficacy of tissue- vs. plasma- vs. plasma plus tissue- NGS of therapeutically targetable mutations in 423 lung cancer patients and also assessed the detection by plasma ctDNA plus tissue NGS to tissue NGS alone.

## Materials and methods

During February 2020 and September 2021, a total of 426 cases of patients with lung cancer who were collected peripheral blood before the treatment and received surgical resection at 56 hospitals were enrolled in this study (Supplemental material). This study was approved by the ethics committees of all the participating hospitals.

All the patients did not receive chemotherapy or radiotherapy before the collection of peripheral blood and surgical resection of tumor tissues, and they had sufficient quality and quantity of tissue DNA for NGS. Diagnosis was based on the morphology of hematoxylin & eosin staining (HE) by two experienced molecular pathologists, and the tumor cell content (tumor purity) was higher than 50%.

For the formalin-fixed paraffin-embedded (FFPE) of tumor tissues, 8–10 of 5–10 μm tumor slices were collected, and blood samples from patients were collected in Ethylene Diamine Tetraacetic Acid (EDTA) tubes for further use. Collected FFPE of tumor tissues and the matched blood samples were then transferred to the laboratory. DNA extraction was used by the DNeasy Blood and Tissue Kit (69,504, QIAGEN, Venlo, Netherlands) according to the manufacturer’s instructions. The content of DNA was determined by Agilent 2100 Bioanalyzer (USA). The targeted libraries were constructed using NGS Fast DNA Library Prep Set (Thermo Fisher, Waltham, MA, USA). DNA sequencing was then performed on Illumina Novaseq 6000 platform according to the manufacturer’s instructions. ZhenXinan ctDNA NGS Panel (Tongshu BioTech, Shanghai, China) adequately covering oncodriver genes targeted NGS was used to identify mutations. All the tumor tissues and peripheral blood (plasma) samples were subjected to NGS of oncodriver mutations. The average sequencing depth in tissues is ≥ 1000×; the average sequencing depth in plasma cfDNA is ≥ 7000×. The variant allele frequency (VAF) is ≥ 1% for tissue DNA and ≥ 0.1% for cfDNA from plasma. BWA (Burrows-Wheeler-Alignment) software was used to compare the sequencing data. GATK (The Genome Analysis Toolkit), M uTect [11] and VarScan [12] were used to alignment optimization, variant calling and annotation, respectively.

Samples which were identified at least one mutation in oncodrivers (*EGFR*, *ERBB2*, *ALK*, *ROS1*, *C-MET*, *KRAS*, *BRAF*, *RET*, *BRCA1* and *BRCA2*) by any of the tissue and plasma assays were considered true positive, and those showed negative by both assays were considered true negative [7]. Concordance was defined as the number of concordant positive and negative cases/total cases × 100%, positive detection rate was calculated as the detected case number/total case number, and sensitivity was shown as the detected case number of true positive/total true positive case number × 100%.

## Results

**Fig. 1:**
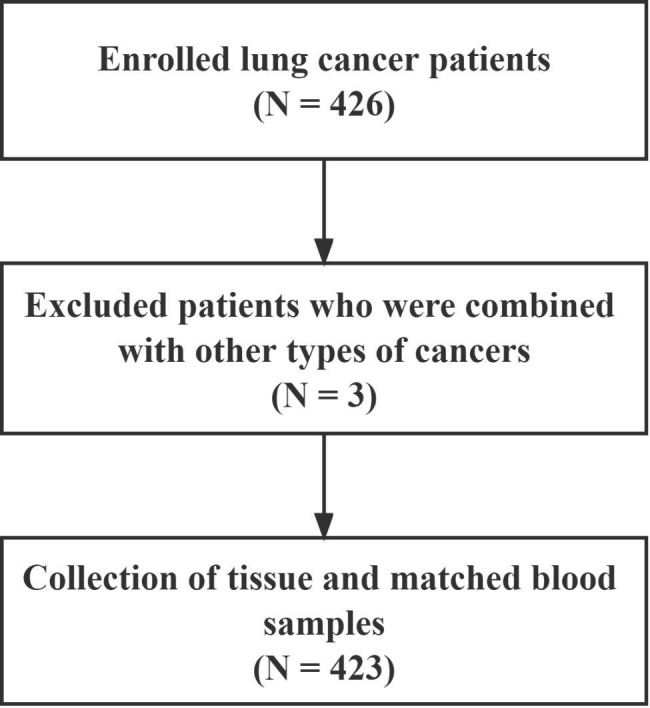
Overview of patient enrollment in this study

A total of 426 patients were enrolled in this study. 3 patients who were combined with other types of cancers were excluded. The flow chart is shown in Fig. 1. Finally, 423 cases of patients with lung cancer were retrospectively reviewed. The basic characteristics of all the cases were presented in Table S1, including 227 males and 196 females, with a median age of 63 years (range, 30–88 years). Regarding histological types, lung adenocarcinoma was the most common subtype (301/423, 71.16%), followed by lung cancer (96/423, 22.69%) and other subtypes (26/423, 6.15%). There were 204 patients at stage IV, 187 at stage III and 32 at stage I-II, 321 patients with low grade cancer and 102 patients with middle grade cancer, and 261 patients with metastasis and 162 without metastasis.

**Fig. 2:**
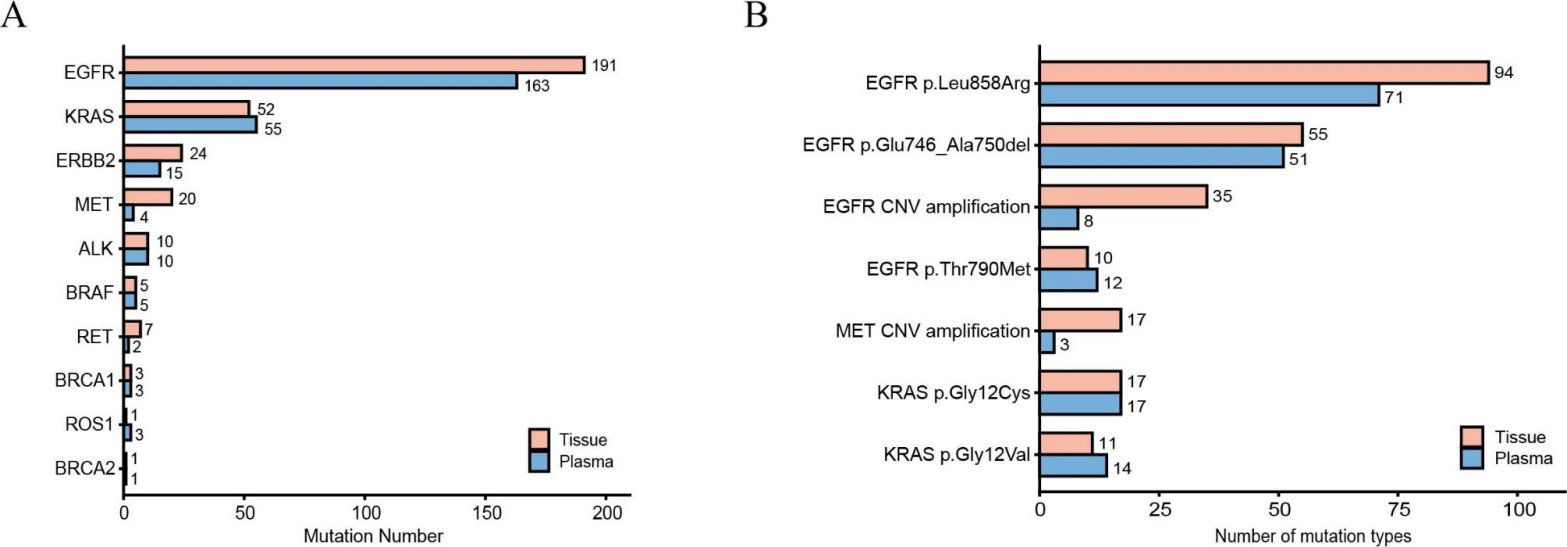
Mutations detected by tissue and plasma ctDNA NGS. (A) Top mutated genes with most mutations detected by tissue and plasma ctDNA NGS, respectively. (B) Top frequent mutations detected by tissue and plasma ctDNA NGS, respectively

35 mutated genes were tested in all the samples of patients (*EGFR*, *TP53*, *FGFR2*, *PTEN*, *RET*, *APC*, *KRAS*, *NRAS*, *BRCA1*, *BRCA2*, *PIK3CA*, *CCND1*, *ERBB2*, *FGFR3*, *FGFR1*, *BRAF*, *CDKN2A*, *CDK4*, *AKT1*, *MET*, *NTRK1*, *DDR2*, *DNMT3A*, *STK11*, *RB1*, *ALK*, *CTNNB1*, *FBXW7*, *CHEK2*, *CCNE1*, *KIT*, *NF1*, *SMARCA4*, *ATM*, *POLE*). A total of 1048 gene mutations were identified in all the samples, of which 605 mutations were identified in tissue samples and 443 were identified in plasma ctDNA samples. In all the 423 patients, no mutations were detected in tissue and matched blood samples of 44 patients. The mutation type of each oncodriver was summarized in Table 1. *EGFR* had the most mutation types (28), followed by *KRAS* (14), *ALK* (9), *ERBB2* (6), etc. In addition, the most mutated oncodrivers were: *EGFR* (191 in tissue vs. 163 in plasma), *KRAS* (52 vs. 55), *ERBB2* (24 vs. 15), *MET* (mainly CNV amplification) (20 vs. 4), *ALK* (mainly *EML-ALK* fusion) (10 vs. 10), *BRAF* (5 vs. 5), *RET* (7 vs. 2), *BRCA1* (3 vs. 3), *ROS1* (1 vs. 3) and *BRCA2* (1 vs. 1) (Fig. 2A, Table S2). The most common types of mutations were *EGFR* p.Leu858Arg (94 in tissue vs. 71 in plasma), *EGFR* p.Glu746_Ala750del (55 vs. 51), *EGFR* CNV amplification (35 vs. 8), *KRAS* p.Gly12Cys (17 vs. 17), *KRAS* p.Gly12Val (11 vs. 14), *EGFR* p.Thr790Met (10 vs. 12), and *MET* CNV amplification (17 vs. 3) (Fig. 2B, Table S2).

**Table 1:** Detected oncogenic mutation types

Mutated genes	Detected mutations
*EGFR* (28)	p.Leu858Arg, p.Glu746_Ala750del, p.Asp770_Pro772dup p.Glu709_Thr710delinsAsp, p.Leu747_Glu749del p.Leu747_Ala750delinsPro, p.Thr790Met, p.Ala767_Val769dup p.Leu861Gln, p.Ser768_Asp770dup, p.Ser752_Ile759del p.Leu747_Pro753delinsSer, p.Glu746_Ala750delinsIlePro p.Glu746_Thr751delinsAla, p.Leu747_Thr751del p.Glu746_Thr751delinsIle, p.Asn771_His773dup, p.Thr751_Ile759delinsAla, p.Glu746_Ser752delinsVal, p.Gly719Ala p.Glu709Lys, p.Gly719Ser, p.Ser768Ile, p.Gly719Cys, p.Glu709Gly p.Leu747_Thr751delinsPro, p.Glu746_Thr751delinsLeu CNV amplification
*KRAS* (14)	p.Gly12Val, p.Gly12Cys, p.Gly12Phe, p.Gln61His, p.Ala146Thr p.Gly12Asp, p.Gln61Leu, p.Gly13Val, p.Gly13Asp, p.Gly12Ala p.Gly13Cys, p.Gly12Ser, p.Gly13Asp GoF, CNV amplification
*ALK* (9)	p.Gly1202Arg, p.Leu1196Met, p.Phe1174Leu, *EML4* (13)-*ALK* (20) fusion *EML4* (14)-*ALK* (19) fusion, *EML4* (6)-*ALK* (20) fusion, *EML4* (21)-*ALK* (20) fusion, *C2orf91* (4)-*ALK* (20) fusion, *KIF5B* (17)-*ALK* (20) fusion
*ERBB2* (6)	p.Tyr772_Ala775dup, p.Gly776delinsValCys, p.Gly778_Pro780dup p.Val777Lue, p.Ser310Phe, CNV amplification
*ROS1* (4)	*CD74* (6)-*ROS1* (34) fusion GoF, *CD74* (7)-*ROS1* (33) fusion *SDC4* (4)-*ROS1* (32) fusion, *EZR* (9)-*ROS1* (33) fusion
*MET* (3)	c.3028 + 1G > T, c.2942-13_2974del, CNV amplification
*BRCA1* (4)	p.Trp1782Ter, p.Thr1691Lys, p.Ser988Ter, c.5332 + 1G > A
*BRCA2* (1)	p.Arg2494Ter
*BRAF* (2)	p.Val600Glu, p.Gly469Ala
*RET* (2)	*CCDC6* (1)-*RET* (12) fusion, *KIF5B* (15)-*RET* (12) fusion

**Fig. 3:**
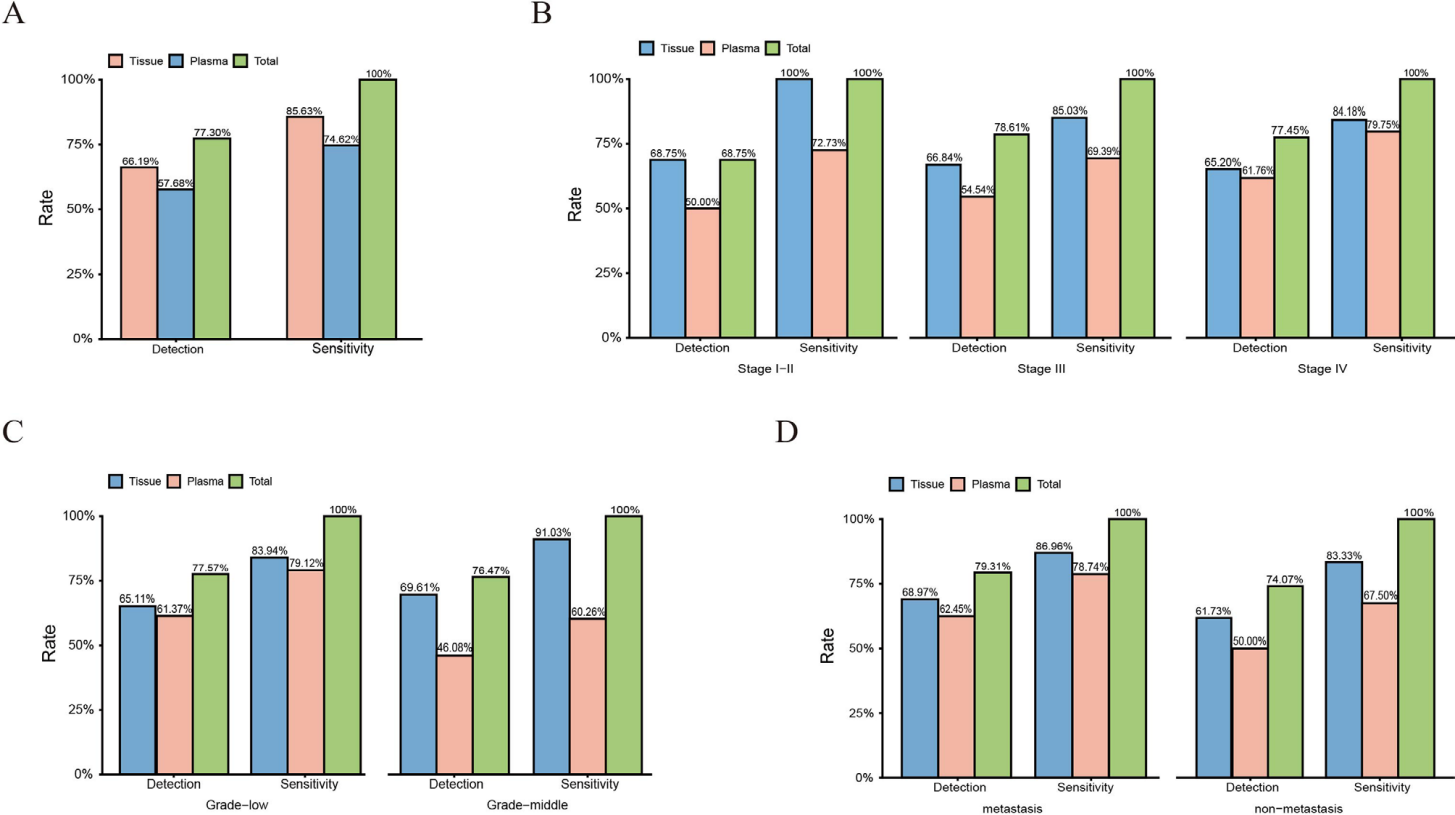
Detection rate and sensitivity for tissue, plasma and the combination in the identification of therapeutically targetable mutations in lung cancer patients and different subpopulations. (A) Detection rate and sensitivity in the whole lung cancer patients. (B) Detection rate and sensitivity in lung cancer patients at stage IV, III and I-II. (C) Detection rate and sensitivity in lung cancer patients with low and middle grades. (D) Detection rate and sensitivity in lung cancer patients with and without metastasis

Among 423 cases, 280 cases showed tissue positive, 242 cases showed plasma positive. In all the 280 positive tissue samples, 364 mutations of oncodrivers were detected, the average number was 1.29. In all the 242 positive plasma samples, 284 mutations of oncodrivers were detected, the average number was 1.16. 197 cases showed both plasma and tissue positive, and 96 showed both negative. The concordance for the positive and negative detection rates between the above two methods were 46.57% (197/423) and 22.70% (96/423), respectively, and total concordance was 69.27% (293/423). Interestingly, 83 (19.62%) cases showed positive by tissue NGS alone (positive in tissues but negative in plasma) and 47 (11.11%) positive by plasma ctDNA alone (positive in plasma but negative in tissues). The total positive case number was 197 + 83 + 47 = 327. The detection rate for tissue, plasma, and plasma plus tissue was (197 + 83)/423 = 66.19%, (197 + 47)/423 = 57.68%, and (197 + 83 + 47)/423 = 77.30%, respectively. Sensitivity of tissue, plasma and plasma plus tissue detection was (197 + 83)/327 = 85.63%, (197 + 47)/327 = 74.62%, and (197 + 83 + 47)/327 = 100.00%, respectively. The result was shown in Tables S3 and Table S4, and Fig. 3A.

There were 204 stage IV, 187 stage III, and 32 I-II patients. As shown in Table S3, Table S4 and Fig. 3B, for 204 stage IV patients, 101 (49.51%) and 46 (22.55%) showed concordant positive and negative in tissue and plasma, respectively. The concordance for detection in tissue and blood was 72.06% (147/204). The detection rates for tissue, plasma and the combination were 65.20%, 61.76% and 77.45%, respectively. The above sensitivity was 84.18%, 79.75%, and 100%, respectively. For 187 stage III patients, the total concordance was 64.17% (120/187), the detection rate was 66.84%, 54.54% and 78.61%, respectively; and sensitivity was 85.03%, 69.39% and 100%, respectively. For the remaining 32 stage I-II cases, the total concordance was 81.25%, detection rates were 68.75%, 50% and 68.75%, respectively, and the sensitivity was 100%, 72.73% and 100%, respectively.

Among 321 low grade patients, the total detection concordance was 71.34% (229/321). Detection rates for tissue, plasma and the combination were 65.11%, 61.37% and 77.57%, respectively; and sensitivity of the above was 83.94%, 79.12% and 100%, respectively. Among the remaining 102 patients with middle grade cancer, the total detection concordance was 62.75% (64/102). Detection rates were 69.61%, 46.08% and 76.47%, respectively; and sensitivity of the above was 91.03%, 60.26% and 100%, respectively (Tables S3, Table S4 and Fig. 3C).

Among 261 patients with metastatic cancer, the total concordance was 72.80% (190/261). Detection rates for tissue, plasma and the combination were 68.97%, 62.45% and 79.31%, respectively; and sensitivity was 86.96%, 78.74% and 100%, respectively. For the remaining 162 patients without metastatic cancer, the total concordance was 63.58% (103/162). Detection rates were 61.73%, 50.0% and 74.07%, respectively; and sensitivity was 83.33%, 67.50% and 100%, respectively (Tables S3, Table S4 and Fig. 3D).

Interestingly, not only in whole-patient population but also in subpopulations classified according to stages, grades and metastatic status, there were some positive cases detected only in tissue and some having therapeutically targetable mutations exclusively detected in plasma (Tables S3 and Table S4).

 [14] [14–16] [17, 18] [19]In addition, we emphatically observed the efficacy between the tissue and plasma ctDNA NGS in the detection of some important clinically relevant mutations, including *EGFR* (containing several main types of mutations), *EGFR* p.Leu858Arg, *EGFR* p.Thr790Met, *EGFR* deletions in exon 19, *ALK* fusion (*EML4-ALK*), *RET* fusion, *ROS1* fusion, *ALK/RET/ROS1* fusion, *MET* gene amplification/variants and *BRAF* p.Val600Glu. It was shown that more mutations could be detected in tissues or plasma, and the combination of the tissue and plasma assays resulted in higher detection rates and sensitivity than any of the two methods (Table 2). In addition, we compared VAF between tumor tissues and plasma ctDNA of these important clinically relevant mutations. The results showed that VAF of some mutations in tumor tissues are significantly higher than those in plasma, e.g. *EGFR* p.Leu858Arg, *KRAS* p.Gly12Cys and ALK (Figure S1; Table S5).

**Table 2:** Important clinically relevant mutations detected by tissue vs. plasma vs. plasma plus tissue-based NGS.

		Tissue+	Tissue-	Total		
*EGFR* (N = 423)	ctDNA+	140	23	163	Tissue sensitivity Tissue detection rate	89.25% 45.15%
	ctDNA-	51	209	260	Plasma sensitivity Plasma detection rate	76.17% 38.53%
	Total	191	232	423	Total detection rate	50.59%
*EGFR* p.Leu858Arg (N = 423)	ctDNA+	67	4	71	Tissue sensitivity Tissue detection rate	95.92% 22.22%
	ctDNA-	27	325	352	Plasma sensitivity Plasma detection rate	72.45% 16.78%
	Total	94	329	423	Total detection rate	23.64%
*EGFR* p.Thr790Met (N = 423)	ctDNA+	8	4	12	Tissue sensitivity Tissue detection rate	71.43% 2.36%
	ctDNA-	2	409	412	Plasma sensitivity Plasma detection rate	85.71% 2.84%
	Total	10	413	423	Total detection rate	3.31%
*EGFR* exon19del (N = 423)	ctDNA+	64	10	74	Tissue sensitivity Tissue detection rate	89.25% 19.62%
	ctDNA-	19	330	349	Plasma sensitivity Plasma detection rate	79.57% 17.49%
	Total	83	340	423	Total detection rate	21.98%
*KRAS* (N = 423)	ctDNA+	38	17	55	Tissue sensitivity Tissue detection rate	75.36% 12.29%
	ctDNA-	14	354	368	Plasma sensitivity Plasma detection rate	79.71% 13.0%
	Total	52	371	423	Total detection rate	16.31%
*KRAS* p.Gly12Cys (N = 423)	ctDNA+	13	4	17	Tissue sensitivity Tissue detection rate	80.95% 4.02%
	ctDNA-	4	402	406	Plasma sensitivity Plasma detection rate	80.95% 4.02%
	Total	17	406	423	Total detection rate	4.96%
*KRAS* p.Gly12Val (N = 423)	ctDNA+	10	4	14	Tissue sensitivity Tissue detection rate	73.33% 2.60%
	ctDNA-	1	408	409	Plasma sensitivity Plasma detection rate	93.33% 3.31%
	Total	11	412	423	Total detection rate	3.55%
*ERBB2* (N = 423)	ctDNA+	12	3	15	Tissue sensitivity Tissue detection rate	88.89% 5.67%
	ctDNA-	12	396	408	Plasma sensitivity Plasma detection rate	55.56% 3.55%
	Total	24	399	423	Total detection rate	6.38%
*MET* (N = 423)	ctDNA+	2	2	4	Tissue sensitivity Tissue detection rate	90.91% 4.73%
	ctDNA-	18	401	419	Plasma sensitivity Plasma detection rate	18.18% 0.95%
	Total	20	403	423	Total detection rate	5.2%
*MET* CNV amplification (N = 423)	ctDNA+	1	2	3	Tissue sensitivity Tissue detection rate	89.47% 4.02%
	ctDNA-	16	404	420	Plasma sensitivity Plasma detection rate	15.79% 0.71%
	Total	17	406	423	Total detection rate	4.49%
*ALK* (N = 423)	ctDNA+	3	7	10	Tissue sensitivity Tissue detection rate	58.82% 2.36%
	ctDNA-	7	406	413	Plasma sensitivity Plasma detection rate	58.82% 2.36%
	Total	10	413	423	Total detection rate	4.02%
*ALK* fusion (N = 423)	ctDNA+	3	5	8	Tissue sensitivity Tissue detection rate	96.97% 91.43%
	ctDNA-	6	409	415	Plasma sensitivity Plasma detection rate	45.45% 42.86%
	Total	9	414	423	Total detection rate	94.29%
*BRAF* (N = 423)	ctDNA+	3	2	5	Tissue sensitivity Tissue detection rate	71.43% 1.18%
	ctDNA-	2	416	418	Plasma sensitivity Plasma detection rate	71.43% 1.18%
	Total	5	418	423	Total detection rate	1.65%
*RET* (N = 423)	ctDNA+	1	1	2	Tissue sensitivity Tissue detection rate	87.5% 1.65%
	ctDNA-	6	415	421	Plasma sensitivity Plasma detection rate	25% 0.47%
	Total	7	416	423	Total detection rate	1.89%
*BRCA1* (N = 423)	ctDNA+	2	1	3	Tissue sensitivity Tissue detection rate	75% 0.71%
	ctDNA-	1	419	420	Plasma sensitivity Plasma detection rate	75% 0.71%
	Total	3	420	423	Total detection rate	0.95%
*ROS1* (N = 423)	ctDNA+	0	3	3	Tissue sensitivity Tissue detection rate	25% 0.24%
	ctDNA-	1	419	420	Plasma sensitivity Plasma detection rate	75% 0.71%
	Total	1	422	423	Total detection rate	0.95%
*BRCA2* (N = 423)	ctDNA+	1	0	1	Tissue sensitivity Tissue detection rate	100% 0.24%
	ctDNA-	0	422	422	Plasma sensitivity Plasma detection rate	100% 0.24%
	Total	1	422	423	Total detection rate	0.24%

## Discussion

*EGFR*, *ERBB2*, *ALK*, *ROS1*, *MET*, *KRAS*, *BRAF*, *RET*, *BRCA1*, *BRCA2*, etc. are important drive genes for the pathogenesis and development of cancers. It is believed that lung cancers are driven by activating or inactivating mutations of multiple oncogenes or tumor suppressor genes, such as *EGFR, KRAS, MET*, and *BRAF*, and translocations in the *ALK, ROS1*, etc. In this study, we comprehensively observed the detection efficacy of tissue- vs. plasma- vs. plasma plus tissue- based NGS of oncodriver mutations as well as compared plasma plus tissue NGS to tissue NGS alone in whole population of 423 lung cancer patients and subpopulations classified according to stages, grades and metastatic status.

The results showed that mutations or CNV amplifications in oncodriver genes were highly occurred in *EGFR, KRAS, ERBB2, MET* (mainly CNV amplification), and *ALK* (mainly *EML-ALK* fusion) by both tissue- and plasma ctDNA- based NGS detection. Moreover, *EGFR, KRAS, MET*, *ERBB2, ALK*, etc. were detected with most mutations, no matter what methods were used. These results showed that both tissue and plasma NGS before systemic therapy are effective in the detection of important oncodriver mutations in lung cancer.

Among a total of 423 cases, concordance for the detection rates of the two methods was 69.27%. The detection rate (57.68%) and sensitivity (74.62%) for plasma were lower than those for tissue (66.19% and 85.63%, respectively). The positive cases in tissues only were more than in plasma only (83 cases vs. 47 cases). In addition, similarly in subpopulations classified according to stages, grades and metastatic status, the detection rate and sensitivity for plasma in different subpopulations were all lower than in tissues, and there were more positive cases detected in tissue only than those in plasma only. These results demonstrated that tissue-NGS is superior to the corresponding plasma ctDNA in the detection of therapeutically targetable mutations (not only the detection rates but also the detected number and types of oncogenic mutations) in lung cancers, regardless of stages, grades and metastatic status, indicating tumor tissue-NGS is a preferred means for molecular profiling of lung cancers as long as tissues are available.

Plasma ctDNA can be conveniently and dynamically used to detect cancer-related gene mutations in peripheral blood to provide basis of precise therapy for cancers, without the necessity of collect solid cancer samples [3–6], while the low content of DNA in blood hampers the wide application of ctDNA. In Schwartzberg et al.’s study, they found that liquid biopsy increases the detection of actionable biomarkers in patients with limited tissue, confirming its value to rescue the patients with tissue unavailable for genomic analysis; however, limited added value of concurrent liquid biopsy in patients that receive tissue comprehensive genomic profiling results [13]. Interestingly, we showed in whole lung cancer patients and subpopulations (patients at different stages, and with different grades and metastatic status), in addition to some positive cases detected only in tissue, there was some percentage of cases (6.86-12.46%) having therapeutically targetable mutations only detected by plasma ctNDA. These results indicated that plasma could additionally capture some therapeutically targetable mutations in lung cancers which might be taken as wild type in tissues, implying that the tissue and plasma detection results should be mutually supplementary.

In a total of 423 cases, although the detection rate and sensitivity of plasma were lower, plasma plus tissue increased the detection of 66.19% by tissue only to 77.30% and sensitivity of 85.63% by tissue only to 100%. Similar results were observed in subpopulations according to different stages (IV vs. III vs. I-II stage), grades (low vs. middle grade) and metastatic status (with vs. without metastasis). These results suggested that plasma ctDNA could effectively enhances the detection rate and sensitivity of therapeutically targetable mutations by tissue only.

Interestingly, the results showed that higher concordance for positive detection rates between tissue- and plasma- NGS in I-II stage/IV vs. III (50%/49.51% vs. 42.78%), low vs. middle grade (48.91% vs. 39.22%), and metastatic vs. non-metastatic lung cancer (52.11% vs. 37.65%). The detection rate and sensitivity for plasma ctDNA was higher in IV vs. III stage, low vs. middle grade, and metastatic vs. non-metastatic cancer. This might be explained that samples from patients at more serious status such as stage IV [14] and low grade and metastasis [15] have more detectable mutations of cancer-related genes.

Our result is inconsistent with some previous studies. For example, Metzenmacher et al. reported a 62.5% mutation positive concordance between tissue-and plasma-NGS in 61 stage IV patients with NSCLC and more somatic variants identified through ctDNA in comparison with the corresponding tumor samples [16]. In contrast, we showed less sensitivity by plasma ctDNA when compared with tissue NGS. This might be related to the difference in genetic characteristics [7], disease status, etc. of the study populations [14], screened oncogenes [17], related techniques, etc. between studies [18]. The content of ctDNA also influence the sensitivity, in a study of Husain et al.’s, the results showed that the sensitivity of liquid biopsy to detect driver alterations identified in tissue biopsy from the same patients ranged from 58 to 86%; however, the sensitivity remained or closed to 100% when ctDNA tumor fraction ≥ 10% [19].

The most common mutations in *EGFR* were deletions in exon 19 as well as p.Leu858Arg as sensitizing mutations, in addition, *EGFR* p.Thr790Met, *EGFR* p.Ser768Ile, etc., are resistance mutations [20]. Through targeting these mutations *EGFR* tyrosine kinase inhibitors (TKIs) drugs have been developed and approved for some NSCLCs carrying related mutations [20–22]. Nevertheless, a second-site mutation of *EGFR* Thr790Met, *MET* gene amplification, etc. confers lung cancer acquire resistance to these EGFR TKIs [23, 24]. In addition, the resultant anaplastic lymphoma kinase (*ALK*) fusion protein with others (*ALK* fusion partners) brings about constitutive *ALK* tyrosine kinase activity mediating oncogenic transformation. The presence of *ALK* fusion, mainly in the form of *EML4-ALK*, in NSCLCs provides clinical beneficial for patients from treatment with *ALK*-directed therapy [25]. Therefore, we also observed the detection efficacy in *EGFR* and main types of mutations which are very important in the tumor response to chemotherapies, targeted and immune therapies, and acquirement of resistance, by tissue- and plasma-NGS. The results showed that *EGFR* p.Leu858Arg, p.Thr790Met, and *EGFR* deletion exon 19 were generally detected more in tissues than in plasma, while in some cases these important mutations were exclusively detected in plasma instead of in the corresponding tissues. This might be attributable to the intratumor heterogeneity in tumor tissues [26], which does not affect plasma ctDNA [27].

These results suggest that to fully capture the clinically targeted mutations in lung cancers, tissue and plasma NGS should be complementary each other. The concept of complementary approach has been proposed by international experts in the field [28] and incorporated in the IASLC Liquid Biopsy statement [29]. In addition, similarly, combination of tissue and plasma methods resulted in higher detection rates and sensitivity in comparison with any of the two methods, further indicating an additional value of plasma ctDNA to tissue NGS in the detection of therapeutically targetable mutations in lung cancers. Furthermore, more and more studies have proposed the concept of turnaround time (TAT), which is defined as the days between test order date and report date [10, 30]. Different studies showed that ctDNA testing is associated with a shorter TAT as compared with tissue testing [10, 31], this time-to-result benefit from plasma-based NGS is particularly important during and following the COVID-19 pandemic, as delays in diagnosis and treatment, are likely to persist for years [32].

There are some limitations in this study. This was a retrospective study that there might be some bias in the section of cases and deficiency of some important information of patients. In addition, in this study only case information was reviewed and no intervention was carried out, therefore the ctDNA/tissue NGS results-guided therapeutic outcomes were not observed and compared. Next, in prospective studies involving more patients, the tissue- and plasma ctDNA NGS-guided therapies will be comparatively observed to further validate this result. Furthermore, because the plasma and tissue based-NGS testing in this study were performed as research, we were not able to capture an accurate measure of TAT. Even with the above limitations, our study still had some of strength. Firstly, our study had a relatively large cohort of 423 patients. Secondly, we comprehensively observed the performance of plasma ctDNA and tumor tissue for detecting the important clinically mutations in from the perspective of the whole population and the subpopulation like grade, stage and metastasis status. Thirdly, our study further verified the importance of plasma-based NGS. Although tissue-based NGS detects significantly more clinically relevant alterations and therapeutic targets, Plasma-based NGS can still play an important role when tissue testing is not possible. When tumor tissue is available, tissue-based NGS should be used in combination with plasma-based NGS to improve detection rate and sensitivity, which could helpful better guide the accurate treatment of lung cancers.

## Conclusion

Plasma ctDNA shares a high concordance with tissue-NGS, and it can additionally capture some important mutations which might be omitted by tissue-NGS. Plasma plus tissue increases the detection rate and sensitivity for tissue NGS only. Tissue and plasma ctDNA NGS sequencing could be mutually complementary to comprehensively provide critical cancer genomic information. This study will be beneficial for the optimal application of plasma ctDNA in the profiling and monitoring of clinically targetable mutations to instruct precise and personalized treatment for lung cancers.

## Electronic supplementary material

Below is the link to the electronic supplementary material.

Supplementary Material 1

Supplementary Material 2

Supplementary Material 3

Supplementary Material 4

Supplementary Material 5

Supplementary Material 6

## Data Availability

We have already uploaded our data on Genome Variation Map repository (Accession number: GVM000474; Submission ID: sub000897; Project number: PRJCA014073) at https://ngdc.cncb.ac.cn/gvm/.

## References

[1] Sung H, Ferlay J, Siegel RL, Laversanne M, Soerjomataram I, Jemal A, Bray F (2021). Global Cancer Statistics 2020: GLOBOCAN estimates of incidence and Mortality Worldwide for 36 cancers in 185 countries. CA Cancer J Clin.

[2] Zappa C, Mousa SA (2016). Non-small cell lung cancer: current treatment and future advances. Transl Lung Cancer Res.

[3] Lebofsky R, Decraene C, Bernard V, Kamal M, Blin A, Leroy Q, Rio Frio T, Pierron G, Callens C, Bieche I (2015). Circulating tumor DNA as a non-invasive substitute to metastasis biopsy for tumor genotyping and personalized medicine in a prospective trial across all tumor types. Mol Oncol.

[4] Wan JCM, Massie C, Garcia-Corbacho J, Mouliere F, Brenton JD, Caldas C, Pacey S, Baird R, Rosenfeld N (2017). Liquid biopsies come of age: towards implementation of circulating tumour DNA. Nat Rev Cancer.

[5] Zill OA, Banks KC, Fairclough SR, Mortimer SA, Vowles JV, Mokhtari R, Gandara DR, Mack PC, Odegaard JI, Nagy RJ (2018). The Landscape of Actionable genomic alterations in cell-free circulating tumor DNA from 21,807 Advanced Cancer Patients. Clin Cancer Res.

[6] Rothwell DG, Ayub M, Cook N, Thistlethwaite F, Carter L, Dean E, Smith N, Villa S, Dransfield J, Clipson A (2019). Utility of ctDNA to support patient selection for early phase clinical trials: the TARGET study. Nat Med.

[7] Lin LH, Allison DHR, Feng Y, Jour G, Park K, Zhou F, Moreira AL, Shen G, Feng X, Sabari J (2021). Comparison of solid tissue sequencing and liquid biopsy accuracy in identification of clinically relevant gene mutations and rearrangements in lung adenocarcinomas. Mod Pathol.

[8] Schouten RD, Vessies DCL, Bosch LJW, Barlo NP, van Lindert ASR, Cillessen S, van den Broek D, van den Heuvel MM, Monkhorst K. Clinical Utility of Plasma-Based Comprehensive Molecular Profiling in Advanced Non-Small-Cell Lung Cancer.JCO Precis Oncol2021, 5. 10.1200/PO.20.00450 PMC8277301 34632253

[9] Aggarwal C, Thompson JC, Black TA, Katz SI, Fan R, Yee SS, Chien AL, Evans TL, Bauml JM, Alley EW (2019). Clinical implications of plasma-based genotyping with the delivery of personalized therapy in metastatic non-small cell Lung Cancer. JAMA Oncol.

[10] Leighl NB, Page RD, Raymond VM, Daniel DB, Divers SG, Reckamp KL, Villalona-Calero MA, Dix D, Odegaard JI, Lanman RB (2019). Clinical utility of Comprehensive Cell-free DNA analysis to identify genomic biomarkers in patients with newly diagnosed metastatic non-small cell Lung Cancer. Clin Cancer Res.

[11] McKenna A, Hanna M, Banks E, Sivachenko A, Cibulskis K, Kernytsky A, Garimella K, Altshuler D, Gabriel S, Daly M (2010). The genome analysis Toolkit: a MapReduce framework for analyzing next-generation DNA sequencing data. Genome Res.

[12] Koboldt DC, Chen K, Wylie T, Larson DE, McLellan MD, Mardis ER, Weinstock GM, Wilson RK, Ding L (2009). VarScan: variant detection in massively parallel sequencing of individual and pooled samples. Bioinformatics.

[13] Schwartzberg LS, Li G, Tolba K, Bourla AB, Schulze K, Gadgil R, Fine A, Lofgren KT, Graf RP, Oxnard GR (2022). Complementary roles for tissue- and blood-based comprehensive genomic profiling for detection of actionable driver alterations in Advanced NSCLC. JTO Clin Res Rep.

[14] Zhang Y, Yao Y, Xu Y, Li L, Gong Y, Zhang K, Zhang M, Guan Y, Chang L, Xia X (2021). Pan-cancer circulating tumor DNA detection in over 10,000 chinese patients. Nat Commun.

[15] Ng CKY, Di Costanzo GG, Tosti N, Paradiso V, Coto-Llerena M, Roscigno G, Perrina V, Quintavalle C, Boldanova T, Wieland S (2018). Genetic profiling using plasma-derived cell-free DNA in therapy-naïve hepatocellular carcinoma patients: a pilot study. Ann Oncol.

[16] Metzenmacher M, Hegedüs B, Forster J, Schramm A, Horn PA, Klein CA, Bielefeld N, Ploenes T, Aigner C, Theegarten D (2022). Combined multimodal ctDNA analysis and radiological imaging for tumor surveillance in non-small cell lung cancer. Transl Oncol.

[17] Bieg-Bourne CC, Okamura R, Kurzrock R (2020). Concordance between TP53 alterations in blood and tissue: impact of time interval, biopsy site, cancer type and circulating tumor DNA burden. Mol Oncol.

[18] Palmero R, Taus A, Viteri S, Majem M, Carcereny E, Garde-Noguera J, Felip E, Nadal E, Malfettone A, Sampayo M (2021). Biomarker Discovery and Outcomes for Comprehensive Cell-Free circulating Tumor DNA Versus Standard-of-care tissue testing in Advanced Non-Small-Cell Lung Cancer. JCO Precis Oncol.

[19] et al: Tumor Fraction Correlates With Detection of Actionable Variants Across > 23,000 Circulating Tumor DNA Samples. *JCO Precis Oncol* 2022, 6:e2200261. 10.1200/PO.22.00261 PMC9616642 36265119

[20] Shi Y, Au JS, Thongprasert S, Srinivasan S, Tsai CM, Khoa MT, Heeroma K, Itoh Y, Cornelio G, Yang PC (2014). A prospective, molecular epidemiology study of EGFR mutations in asian patients with advanced non-small-cell lung cancer of adenocarcinoma histology (PIONEER). J Thorac Oncol.

[21] Shi YK, Wang L, Han BH, Li W, Yu P, Liu YP, Ding CM, Song X, Ma ZY, Ren XL (2017). First-line icotinib versus cisplatin/pemetrexed plus pemetrexed maintenance therapy for patients with advanced EGFR mutation-positive lung adenocarcinoma (CONVINCE): a phase 3, open-label, randomized study. Ann Oncol.

[22] Wu YL, Cheng Y, Zhou X, Lee KH, Nakagawa K, Niho S, Tsuji F, Linke R, Rosell R, Corral J (2017). Dacomitinib versus gefitinib as first-line treatment for patients with EGFR-mutation-positive non-small-cell lung cancer (ARCHER 1050): a randomised, open-label, phase 3 trial. Lancet Oncol.

[23] Sequist LV, Waltman BA, Dias-Santagata D, Digumarthy S, Turke AB, Fidias P, Bergethon K, Shaw AT, Gettinger S, Cosper AK (2011). Genotypic and histological evolution of lung cancers acquiring resistance to EGFR inhibitors. Sci Transl Med.

[24] Yu HA, Arcila ME, Rekhtman N, Sima CS, Zakowski MF, Pao W, Kris MG, Miller VA, Ladanyi M, Riely GJ (2013). Analysis of tumor specimens at the time of acquired resistance to EGFR-TKI therapy in 155 patients with EGFR-mutant lung cancers. Clin Cancer Res.

[25] Koivunen JP, Mermel C, Zejnullahu K, Murphy C, Lifshits E, Holmes AJ, Choi HG, Kim J, Chiang D, Thomas R (2008). EML4-ALK fusion gene and efficacy of an ALK kinase inhibitor in lung cancer. Clin Cancer Res.

[26] Ramón YCS, Sesé M, Capdevila C, Aasen T, De Mattos-Arruda L, Diaz-Cano SJ, Hernández-Losa J, Castellví J (2020). Clinical implications of intratumor heterogeneity: challenges and opportunities. J Mol Med (Berl).

[27] Nong J, Gong Y, Guan Y, Yi X, Yi Y, Chang L, Yang L, Lv J, Guo Z, Jia H (2018). Circulating tumor DNA analysis depicts subclonal architecture and genomic evolution of small cell lung cancer. Nat Commun.

[28] Aggarwal C, Rolfo CD, Oxnard GR, Gray JE, Sholl LM, Gandara DR (2021). Strategies for the successful implementation of plasma-based NSCLC genotyping in clinical practice. Nat Rev Clin Oncol.

[29] Rolfo C, Mack P, Scagliotti GV, Aggarwal C, Arcila ME, Barlesi F, Bivona T, Diehn M, Dive C, Dziadziuszko R (2021). Liquid Biopsy for Advanced NSCLC: a Consensus Statement from the International Association for the study of Lung Cancer. J Thorac Oncol.

[30] Cui W, Milner-Watts C, O’Sullivan H, Lyons H, Minchom A, Bhosle J, Davidson M, Yousaf N, Scott S, Faull I (2022). Up-front cell-free DNA next generation sequencing improves target identification in UK first line advanced non-small cell lung cancer (NSCLC) patients. Eur J Cancer.

[31] Mack PC, Banks KC, Espenschied CR, Burich RA, Zill OA, Lee CE, Riess JW, Mortimer SA, Talasaz A, Lanman RB (2020). Spectrum of driver mutations and clinical impact of circulating tumor DNA analysis in non-small cell lung cancer: analysis of over 8000 cases. Cancer.

[32] National Health Service England. Waiting times for suspected and diagnosed cancer patients. *2020-21 annual report* 2021.

