# Supplementary material for: Plasma ctDNA increases tissue NGS-based detection of therapeutically targetable mutations in lung cancers
Source: BMC Cancer. 2023 Mar 31;23:294. doi: 10.1186/s12885-023-10674-z (PMC10063947; doi:10.1186/s12885-023-10674-z)
Supplement: Supplementary file 2 — Supplementary Material 2 [file 12885_2023_10674_MOESM2_ESM.docx]

**Table S2. Oncogenic mutations and mutation types detected in tissue *vs* plasma.**

| **Mutated gene** | **Mutation number** | **Tissue/plasma** |
| --- | --- | --- |
| *EGFR* | 191 | Tissue |
|  | 163 | Plasma |
| *EGFR* p.Leu858Arg | 94 | Tissue |
|  | 71 | Plasma |
| *EGFR* p.Glu746_Ala750del | 55 | Tissue |
|  | 51 | Plasma |
| *EGFR* CNV amplification | 35 | Tissue |
|  | 8 | Plasma |
| *EGFR* p.Thr790Met | 10 | Tissue |
|  | 12 | Plasma |
| *KRAS* | 52 | Tissue |
|  | 55 | Plasma |
| *KRAS* p.Gly12Cys | 17 | Tissue |
|  | 17 | Plasma |
| *KRAS* p.Gly12Val | 11 | Tissue |
|  | 14 | Plasma |
| *ERBB2* | 24 | Tissue |
|  | 15 | Plasma |
| *MET* | 20 | Tissue |
|  | 4 | Plasma |
| *ALK* | 10 | Tissue |
|  | 10 | Plasma |
| *BRAF* | 5 | Tissue |
|  | 5 | Plasma |
| *RET* | 7 | Tissue |
|  | 2 | Plasma |
| *BRCA1* | 3 | Tissue |
|  | 3 | Plasma |
| *ROS1* | 1 | Tissue |
|  | 3 | Plasma |
| *BRCA2* | 1 | Tissue |
|  | 1 | Plasma |
