# Supplementary material for: Plasma ctDNA increases tissue NGS-based detection of therapeutically targetable mutations in lung cancers
Source: BMC Cancer. 2023 Mar 31;23:294. doi: 10.1186/s12885-023-10674-z (PMC10063947; doi:10.1186/s12885-023-10674-z)
Supplement: Supplementary file 4 — Supplementary Material 4 [file 12885_2023_10674_MOESM4_ESM.docx]

**Table S4.** **Detection of plasma-based, tissue-based, and plasma plus** **tissue-based NGS of therapeutically targetable mutations in whole lung cancer patients and subpopulations classified according to stages, grades and metastatic status.**

|  |  | Tissue+ | Tissue- | Total |  |  |  |  |
| --- | --- | --- | --- | --- | --- | --- | --- | --- |
| Whole population  (N=423) | ctDNA+ | 197 | 47 | 244 | Concordance for positive detection in tissue & plasma | 46.57% | Tissue sensitivity  Tissue detection rate | 85.63%  66.19% |
|  | ctDNA- | 83 | 96 | 179 | Concordance for negative detection in tissue & plasma | 22.70% | Plasma sensitivity  Plasma detection rate | 74.62%  57.68% |
|  | Total | 280 | 143 | 423 | Total concordance | 69.27% | Total detection rate | 77.30% |
| Stage IV  (N=204) | ctDNA+ | 101 | 25 | 126 | Concordance for positive detection in tissue & plasma | 49.51% | Tissue sensitivity  Tissue detection rate | 84.18%  65.20% |
|  | ctDNA- | 32 | 46 | 78 | Concordance for negative detection in tissue & plasma | 22.55% | Plasma sensitivity  Plasma detection rate | 79.75%  61.76% |
|  | Total | 133 | 71 | 204 | Total concordance | 72.06% | Total detection rate | 77.45% |
| Stage III  (N=187) | ctDNA+ | 80 | 22 | 102 | Concordance for positive detection in tissue & plasma | 42.78% | Tissue sensitivity  Tissue detection rate | 85.03%  66.84% |
|  | ctDNA- | 45 | 40 | 85 | Concordance for negative detection in tissue & plasma | 21.39% | Plasma sensitivity  Plasma detection rate | 69.39%  54.54% |
|  | Total | 125 | 62 | 187 | Total concordance | 64.17% | Total detection rate | 78.61% |
| Stage I-II  (N=32) | ctDNA+ | 16 | 0 | 16 | Concordance for positive detection in tissue & plasma | 50% | Tissue sensitivity  Tissue detection rate | 100%  68.75% |
|  | ctDNA- | 6 | 10 | 16 | Concordance for negative detection in tissue & plasma | 31.25% | Plasma sensitivity  Plasma detection rate | 72.73%  50% |
|  | Total | 22 | 10 | 32 | Total concordance | 81.25% | Total detection rate | 68.75% |
| Low grade  (N=321) | ctDNA+ | 157 | 40 | 197 | Concordance for positive detection in tissue & plasma | 48.91% | Tissue sensitivity  Tissue detection rate | 83.94%  65.11% |
|  | ctDNA- | 52 | 72 | 124 | Concordance for negative detection in tissue & plasma | 22.43% | Plasma sensitivity  Plasma detection rate | 79.12%  61.37% |
|  | Total | 209 | 112 | 321 | Total concordance | 71.34% | Total detection rate | 77.57% |
| Middle grade  (N=102) | ctDNA+ | 40 | 7 | 47 | Concordance for positive detection in tissue & plasma | 39.22% | Tissue sensitivity  Tissue detection rate | 91.03%  69.61% |
|  | ctDNA- | 31 | 24 | 55 | Concordance for negative detection in tissue & plasma | 23.53% | Plasma sensitivity  Plasma detection rate | 60.26%  46.08% |
|  | Total | 71 | 31 | 102 | Total concordance | 62.75% | Total detection rate | 76.47% |
| Metastasis  (N=261) | ctDNA+ | 136 | 27 | 163 | Concordance for positive detection in tissue & plasma | 52.11% | Tissue sensitivity  Tissue detection rate | 86.96%  68.97% |
|  | ctDNA- | 44 | 54 | 98 | Concordance for negative detection in tissue & plasma | 20.69% | Plasma sensitivity  Plasma detection rate | 78.74%  62.45% |
|  | Total | 180 | 81 | 261 | Total concordance | 72.80% | Total detection rate | 79.31% |
| Non-metastasis  (N=162) | ctDNA+ | 61 | 20 | 81 | Concordance for positive detection in tissue & plasma | 37.65% | Tissue sensitivity  Tissue detection rate | 83.33%  61.73% |
|  | ctDNA- | 39 | 42 | 81 | Concordance for negative detection in tissue & plasma | 25.93% | Plasma sensitivity  Plasma detection rate | 67.50%  50% |
|  | Total | 100 | 62 | 162 | Total concordance | 63.58% | Total detection rate | 74.07% |
