# Supplementary material for: Plasma ctDNA increases tissue NGS-based detection of therapeutically targetable mutations in lung cancers
Source: BMC Cancer. 2023 Mar 31;23:294. doi: 10.1186/s12885-023-10674-z (PMC10063947; doi:10.1186/s12885-023-10674-z)
Supplement: Supplementary file 6 — Supplementary Material 6 [file 12885_2023_10674_MOESM6_ESM.docx]

**
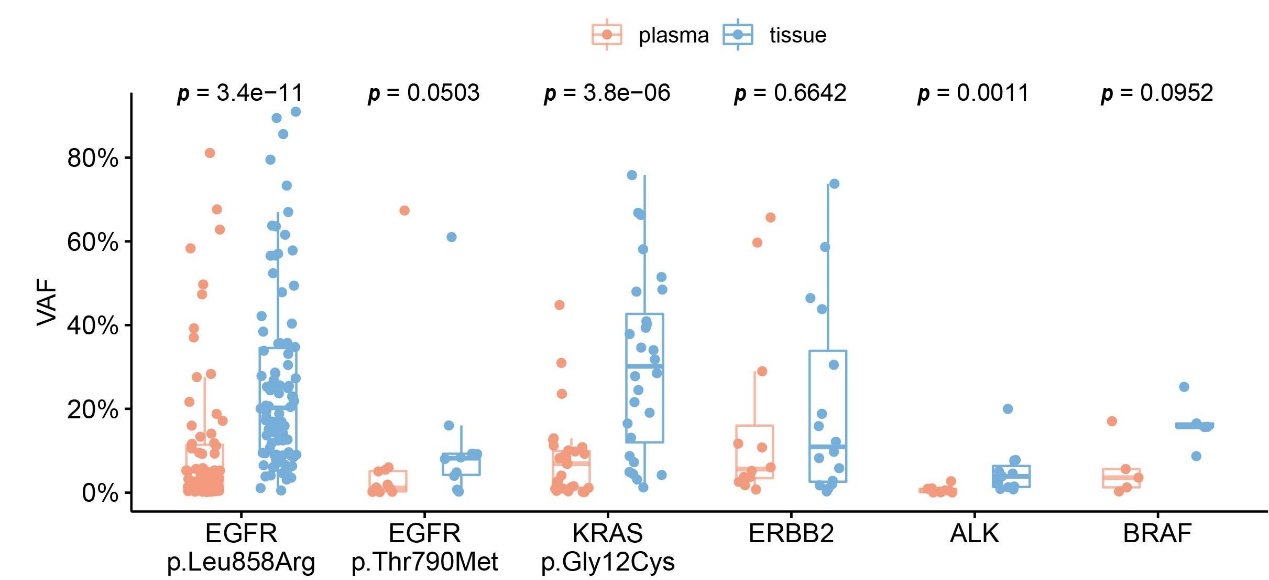
Figure S1.** Comparison of differences between tumor tissues and plasma ctDNA VAF of the important clinically relevant mutations.
